# Postmortem Surveillance for Ebola Virus Using OraQuick Ebola Rapid Diagnostic Tests, Eastern Democratic Republic of the Congo, 2019–2020

**DOI:** 10.3201/eid2802.210981

**Published:** 2022-02

**Authors:** Daniel Mukadi-Bamuleka, Yibayiri Osee Sanogo, Junior Bulabula-Penge, Maria E. Morales-Betoulle, Patrice Fillon, Patrick Woodruff, Mary J. Choi, Amy Whitesell, Alison M. Todres, Anja De Weggheleire, Anaïs Legand, Jean-Jacques Muyembe-Tamfum, Pierre Formenty, John D. Klena, Joel M. Montgomery, Steve Ahuka-Mundeke, RDT Working Group

**Affiliations:** Institut National de Recherche Biomédicale, Kinshasa, Democratic Republic of the Congo (D. Mukadi-Bamuleka, J. Bulabula-Penge, J. Muyembe-Tamfum, S. Ahuka-Mundekea);; Kinshasa Teaching School of Medicine, University of Kinshasa, Kinshasa (D. Mukadi-Bamuleka, J. Bulabula-Penge, J. Muyembe-Tamfum, S. Ahuka-Mundekea);; US Centers for Disease Control and Prevention, Atlanta, Georgia, USA (Y.O. Sanogo, Maria E. Morales-Betoulle, M.J. Choi, A. Whitesell, A.M. Todres, J.D. Klena, J.M. Montgomery);; iMMAP, Geneva, Switzerland (P. Fillon);; Family Health International 360 (FHI360), Crisis Response DRC, Democratic Republic of the Congo (P. Woodruff);; Institute of Tropical Medicine, Antwerp, Belgium (A. De Weggheleire);; World Health Organization Health Emergencies Programme, Geneva, Switzerland (A. Legand, P. Formenty)

**Keywords:** Ebola virus, rapid diagnostic test, postmortem surveillance, Democratic Republic of the Congo, OraQuik Ebola RDT, viruses, zoonoses

## Abstract

After a pilot study, we tested 443 cadavers using OraQuick Ebola rapid diagnostic tests during surveillance after the 10th Ebola outbreak in the Democratic Republic of the Congo. No false negative and 2% false-positive results were reported. Quickly returning results and engaging the community enabled timely public health actions.

The 10th outbreak of Ebola virus (EBOV) disease (EVD) in North Kivu, Democratic Republic of the Congo (DRC), was the longest (August 1, 2018–July 25, 2020) and largest EVD outbreak in DRC; 2,287 persons died and 1,171 survived. A case of EVD recrudescence (recorded June 15, 2019) resulted in 91 additional infections in 6 health zones (*1*–*3*).

Challenges in controlling this EVD outbreak included security threats, widespread community mistrust in response activities, and low acceptance of systematic safe and dignified burials (SDBs). The difficulty with SDBs was in part because of long turnaround times (4 h to 72 h) of required quantitative reverse transcription PCR (RT-PCR) results for burial to bereaved families.

During the postepidemic period, enhanced surveillance of EVD is critical for controlling outbreaks because of potential flare-ups from undetected transmission chains or recrudescence in survivors (*4*–*7*). The objective of this study was to strengthen laboratory surveillance by quickly returning test results to families for timely public health interventions and to improve community engagement and acceptance of SDBs. After a pilot study conducted during active EVD transmission, we used OraQuick Ebola rapid diagnostic tests (RDTs; OraSure Technologies, Inc., https://www.orasure.com) to screen for EBOV infection in decedents within the community and in healthcare facilities during the postepidemic enhanced surveillance period using real-time field data reporting and molecular confirmation.

## The Study

OraQuick Ebola is the first RDT licensed by the US Food and Drug Administration for EVD screening using blood or cadaver fluid samples (*8*). The US Centers for Disease Control and Prevention and the World Health Organization have recommended its use for testing cadaver fluids of suspected EVD patients (*9*). Ethics approval and participant consent were not deemed necessary because specimens were collected for outbreak response and data were de-identified before analysis. A consortium of laboratory, epidemiology, communication, and community engagement professionals, led by the DRC Ministry of Health, formed an RDT technical working group to coordinate field implementation, including SDBs, community engagement, and data collection. A steering committee composed of senior leaders from the Institut National de Recherche Biomédicale (INRB) and international partners advised the RDT Working Group.

The pilot study was conducted during active EBOV transmission (October 31–December 31, 2019) in Mambasa, Mandima, and Beni health zones. Trained healthcare workers conducted RDTs in communities and healthcare facilities. Data were collected manually. Samples were shipped to the INRB lab for confirmation by RT-PCR. SDBs were systematically performed on all cadavers regardless of RDT results. Some community reticence was encountered during the pilot study; violence led to change of location from Mambasa to Beni.

Of 196 cadavers tested by RDTs during the pilot study, 12 (6%) were reactive, of which 4 were negative by RT-PCR (2% false positive) (Table 1). Positive predictive value was 66% and negative predictive value 100% (no false negatives). Among confirmed cases, EBOV gene cycle thresholds ranged from 15.8 to 27.7 for nucleoprotein and 12.3 to 31.4 for glycoprotein. Lessons learned from the pilot study included the need for better community engagement, improved data collection and reporting, and more in-depth healthcare worker training.

**Table 1:** Summary results of RDT pilot study performed on cadaver oral fluid in Mambasa, Mandima, and Beni health zones during active transmission of Ebola virus, DRC, 2019–2020*

RDT results	PCR results		Total
Confirmed	Not confirmed
Reactive	8	4	12
Nonreactive	0	182	182	
Invalid	0	2	2	
Total	8	188	196

*DRC, Democratic Republic of the Congo; RDT, rapid diagnostic test (OraQuick, OraSure Technologies, Inc., https://www.orasure.com).

**Figure 1:**
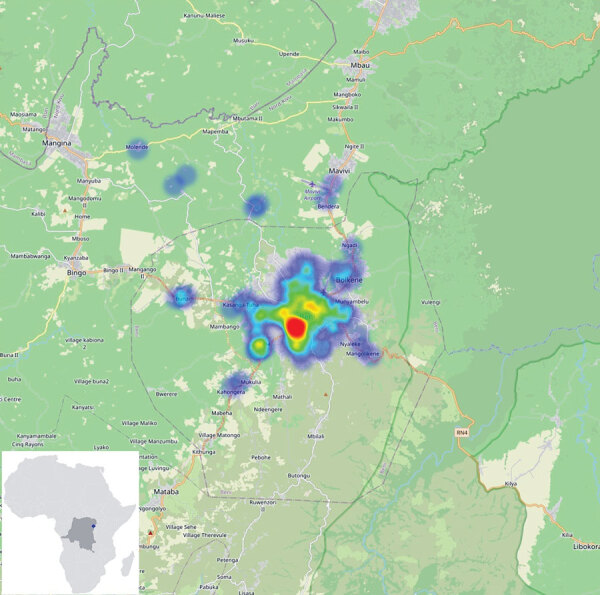
Beni Health zone with sites of Ebola virus disease sample collection for study on postmortem surveillance for Ebola virus using OraQuick (OraSure Technologies, Inc., https://www.orasure.com) Ebola rapid diagnostic tests, eastern Democratic Republic of the Congo, 2019–2020. The numbers and the geolocation rapid diagnostic testing are provided in heatmaps from blue (fewer cases) to red (most cases). Most of the cases were from the health care facilities in Beni township. Inset shows location of the Beni Health zone in the Democratic Republic of the Congo and in Africa.

**Figure 2:**
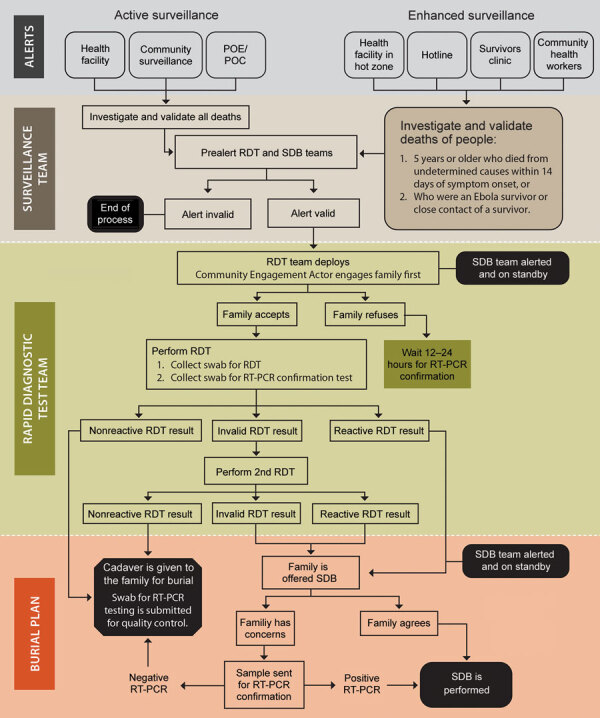
Algorithm of Ebola virus disease RDT implementation in North Kivu in the Beni health zone during active transmission (active surveillance) and postepidemic enhanced surveillance (enhanced surveillance), Democratic Republic of the Congo, 2019–2020. This information was used to inform burial planning and SDBs when indicated. EVD, Ebola virus disease; RDT, rapid diagnostic test (OraQuick, OraSure Technologies, Inc., https://www.orasure.com); RT-PCR, reverse transcription PCR; SDBs, safe and dignified burials.

After the pilot study, RDT postepidemic (August 1–October 31, 2020) surveillance was conducted on cadavers in 19 health areas of the Beni health zone (Figure 1), the last health zone affected during the outbreak. RDTs were administered by 32 teams of locally trained healthcare workers, each composed of a laboratorian or nurse, a hygienist, a community engagement specialist, and a supervisor. The laboratorian/nurse collected 1 swab sample with the pad of the OraQuick device for the RDT and stored another swab sample in viral transport medium for quantitative RT-PCR confirmation. The hygienist oversaw biosafety practices and ensured that biologic waste (used RDT kits and personal protective equipment) was properly incinerated. A community engagement specialist communicated with the family, provided psychosocial support, and engaged the community using media and interactions with local leaders. The supervisor assumed responsibility for RDT quality control. Field teams were provided with the testing algorithm (Figure 2), a field training manual*,* and communication materials to assist with community engagement. SDB teams were on standby for safe burials as requested by families or if a sample was reactive/invalid.

Data were collected using tablets outfitted with a free, open-source, Kobo–based mobile data collection tool (https://www.humanitarianresponse.info/fr/applications/kobotoolbox.com), developed for this purpose using a set of 75 questions in French. The data collection tool operated offline. RDT data, collection site geolocations, and photographs of RDT results were transmitted daily to the Kobo server when internet connection was available. A dashboard displaying key indicators was updated automatically twice a day. We used R software (*10*) to assess the diagnostic accuracy of the RDTs, using quantitative RT-PCR results as the standard.

After receiving permission from decedents’ families, the laboratorian/nurse hygienist performed the test following instructions in the manual (S2). Results were read, interpreted, and photographed at 30 minutes, according to the manufacturer’s instructions. If the RDT was nonreactive, families could proceed with traditional burial. If the RDT was reactive or invalid, the sample in viral transport medium, packaged in cooler boxes with ice packs, was transported immediately to an INRB lab for confirmation by GeneXpert Ebola quantitative RT-PCR (Cepheid, https://www.cepheid.com), with result turnaround time under 24 hours. An RDT was considered invalid when, after 1 repeat, no line appeared in the C area of the test, a purple background obscured the results, or a partial line appeared in the C or T area after 30 minutes.

During postepidemic surveillance, 443 cadavers were tested (3 cadavers were removed by families before RDTs were performed): 235 (53%) were from mortuaries, 111 (25%) from the community, and 97 (22%) from hospitals. Swab specimens were collected from 272 (61%) male and 171 (39%) female cadavers; 27% were children <5 years. Of the 443 samples, 425 (96%) had nonreactive RDTs, 11 (2%) were invalid, and 7 (2%) were reactive. Reactive, invalid and nonreactive samples tested by quantitative RT-PCR (363) were all negative, yielding 6 false-positive and no false-negative results (Table 2). One reactive RDT was not confirmed by quantitative RT-PCR. Although no EVD cases were confirmed among decedents, 32 SDBs were requested by families.

**Table 2:** Summary results of RDTs performed on cadaver oral fluids in the Beni health zone during the 90-day enhanced surveillance period after 10th EVD outbreak in DRC, 2019–2020*

RDT results	PCR results			
	Positive	Negative	Not done	RDT totals
Reactive	0	6	1	7
Non-reactive	0	348	77	425	
Invalid	0	9	2	11	
PCR total	0	363	80	443

*Fifteen RDTs performed to retest invalid and nonreactive initial RDTs are not included. DRC, Democratic Republic of the Congo; EVD, Ebola virus disease; RDT, rapid diagnostic test (OraQuick, OraSure Technologies, Inc., https://www.orasure.com).

## Conclusions

Trained local healthcare workers successfully used OraQuick Ebola RDTs for enhanced postmortem surveillance after the 10th EVD outbreak in DRC. Molecular testing revealed no false-negative RDT results, suggesting that quick public health actions can be based on RDT results alone. The low cycle thresholds observed in positive samples during the pilot study (Appendix Table) support using RDTs in cadavers, in which viral loads are expected to be high (*11*–*13*). Our study shows that RDTs can detect EVD-related deaths and reduce the risk for community transmission. The utility of this tool in EVD surveillance is supported by recent observations that SDBs were not conducted during early stages of recent EVD resurgences in North Kivu and Guinea (CDC 2021 Ebola Response, unpub. data).

In conclusion, postmortem OraQuick Ebola RDTs effectively complemented outbreak-response efforts, improved community trust, and decreased the number of SDBs. However, the reported 2% false-positive tests required further confirmation and were not immediately actionable. SDBs requested by families despite nonreactive RDT further highlight the need for further community engagement.

Appendix Additional information on postmortem surveillance for Ebola virus using OraQuick Ebola rapid diagnostic tests, eastern Democratic Republic of the Congo, 2019–2020.
