# Supplementary material for: Postmortem Surveillance for Ebola Virus Using OraQuick Ebola Rapid Diagnostic Tests, Eastern Democratic Republic of the Congo, 2019–2020
Source: Emerg Infect Dis. 2022 Feb;28(2):420–4. doi: 10.3201/eid2802.210981 (PMC8798676; doi:10.3201/eid2802.210981)
Supplement: Appendix — Additional information on postmortem surveillance for Ebola virus using OraQuick Ebola rapid diagnostic tests, eastern Democratic Republic of the Congo, 2019–2020. [file 21-0981-Techapp-s1.pdf]

Article DOI: https://doi.org/10.3201/eid2802.210981

Postmortem Surveillance for Ebola Virus Using OraQuick Ebola Rapid Diagnostic Tests, Eastern Democratic Republic of the Congo, 2019–2020

Appendix

Appendix Table. Epidemiologic information and results of quantitative reverse transcription PCR of reactive results of suspected Ebola samples during postmortem testing with Oraquick tests

Sample	Age/sex	Health area	Health zone	Sampling date, 2019	Epidemiologic link	Oraquick Ebola	Gene Xpert ® Ebola			
							NP	CT (NP)	GP	CT (GP)
Death 1	–/F*	Some	Mambasa	Nov 14	Yes	Reactive	Negative	0	Negative	0
Death 2	19/F	Lwemba	Mandima	Nov 23	No	Reactive	Positive	25.8	Positive	21.0
Death 3	20/M	Biakato-mine	Mandima	Nov 27	Yes	Reactive	Positive	18.1	Positive	22.9
Death 4	22/F	Lwemba	Mandima	Nov 27	Yes	Reactive	Positive	24.1	Positive	19.7
Death 5	1/M	Biakato-mine	Mandima	Nov 30	Yes	Reactive	Negative	0	Negative	0
Death 6	0/M	Biakato-mine	Mandima	Dec 2	Yes	Reactive	Negative	0	Negative	0
Death 7	20/F	Masosi	Oicha	Dec 4	Yes	Reactive	Positive	27.7	Positive	31.4
Death 8	44/M	Lwemba	Mandima	Dec 5	No	Reactive	Positive	23.7	Positive	19.5
Death 9	22/F	Aloya	Mabaloko	Dec 6	Yes	Reactive	Positive	23.6	Positive	19.9
Death 10	4/M	Aloya	Mabaloko	Dec 22	Yes	Reactive	Positive	15.8	Positive	12.3
Death 11	1/F	Aloya	Mabaloko	Dec 23	No	Reactive	Positive	20.9	Positive	16.2
Death 12	65/F	†	Oicha	Dec 23	No	Reactive	Negative	0	Negative	0

*Unknown age
†Unknown health area.
